# The gut symbiont *Sphingomonas* mediates imidacloprid resistance in the important agricultural insect pest *Aphis gossypii* Glover

**DOI:** 10.1186/s12915-023-01586-2

**Published:** 2023-04-17

**Authors:** Nannan Lv, Ren Li, Shenhang Cheng, Lei Zhang, Pei Liang, Xiwu Gao

**Affiliations:** Department of Entomology, China Agricultural University, Beijing, 100193 China

**Keywords:** *Aphid gossypii*; Insecticide resistance; Symbiotic bacteria; *Sphingomonas*

## Abstract

**Background:**

Neonicotinoid insecticides are applied worldwide for the control of agricultural insect pests. The evolution of neonicotinoid resistance has led to the failure of pest control in the field. The enhanced detoxifying enzyme activity and target mutations play important roles in the resistance of insects to neonicotinoid resistance. Emerging evidence indicates a central role of the gut symbiont in insect pest resistance to pesticides. Existing reports suggest that symbiotic microorganisms could mediate pesticide resistance by degrading pesticides in insect pests.

**Results:**

The 16S rDNA sequencing results showed that the richness and diversity of the gut community between the imidacloprid-resistant (IMI-R) and imidacloprid-susceptible (IMI-S) strains of the cotton aphid *Aphis gossypii* showed no significant difference, while the abundance of the gut symbiont *Sphingomonas* was significantly higher in the IMI-R strain. Antibiotic treatment deprived *Sphingomonas* of the gut, followed by an increase in susceptibility to imidacloprid in the IMI-R strain. The susceptibility of the IMI-S strain to imidacloprid was significantly decreased as expected after supplementation with *Sphingomonas*. In addition, the imidacloprid susceptibility in nine field populations, which were all infected with *Sphingomonas*, increased to different degrees after treatment with antibiotics. Then, we demonstrated that *Sphingomonas* isolated from the gut of the IMI-R strain could subsist only with imidacloprid as a carbon source. The metabolic efficiency of imidacloprid by *Sphingomonas* reached 56% by HPLC detection. This further proved that *Sphingomonas* could mediate *A. gossypii* resistance to imidacloprid by hydroxylation and nitroreduction.

**Conclusions:**

Our findings suggest that the gut symbiont *Sphingomonas*, with detoxification properties, could offer an opportunity for insect pests to metabolize imidacloprid. These findings enriched our knowledge of mechanisms of insecticide resistance and provided new symbiont-based strategies for control of insecticide-resistant insect pests with high *Sphingomonas* abundance.

**Supplementary Information:**

The online version contains supplementary material available at 10.1186/s12915-023-01586-2.

## Background

Neonicotinoid insecticides have been applied worldwide, and by 2015, they accounted for approximately 25% of total global insecticide sales [1]. As selective agonists of insect nicotinic acetylcholine receptors (nAChRs), neonicotinoids are very effective in controlling sucking pests and several coleopteran, dipteran, and lepidopteran pest species [2]. Imidacloprid, which was launched in 1991 by Bayer Crop Science, was the first neonicotinoid insecticide. Due to its low mammalian toxicity, imidacloprid (IMI) has replaced many carbamates and organophosphates in crop protection [3]. It was reported that imidacloprid failed to control the cotton whitefly, *Bemisia tabaci*, in Spain, the first field failure of neonicotinoids worldwide [4]. With the widespread use of imidacloprid, issues of imidacloprid resistance are often reported. It has been detected that the resistance ratio of field populations of *Nilaparvata lugens* to imidacloprid has been up to 248-fold in Thailand [5]. In China, imidacloprid was applied to control *Aphis gossypii* in most regions, and high selection pressure has resulted in high imidacloprid resistance in this pest [6, 7].

The cotton aphid, *A. gossypii* (Hemiptera: Aphididae), is a sap-feeding insect species that can cause considerable economic damage to cotton and numerous other crops worldwide [8]. With the widespread cultivation of transgenic *Bt* cotton, major devastating lepidopteran pests, such as *Helicoverpa armigera*, have been successfully controlled [9]. The occurrence of secondary pests such as *A. gossypii* increased and gradually increased to become the main pests in the cotton fields of China [10]. Some research has shown that the resistance mechanisms of insect pests to neonicotinoids are related to an increase in detoxification enzyme activity and/or mutations in the nicotinic acetylcholine receptor (nAChR) molecular targets [11, 12]. Cytochrome P450-mediated detoxification might play a primary role in insect resistance to neonicotinoids [13]. *CYP6CY22* and *CYP6CY13* of cytochrome P450s in *A. gossypii* could metabolize imidacloprid, which explained its high resistance level to imidacloprid [14]. The overexpression of uridine 5-diphosphate glucuronosyltransferases (UGTs) was also found to be associated with imidacloprid resistance in field populations of *A. gossypii* [15]. Another important mechanism of insect resistance to neonicotinoids is mutations in the *β*1 subunit of the nAChR. The mutation R81T was reported by Koo et al. [16], and then the mutations L80S [17], V62I, and K264E [7] were reported successively. Some researchers have shown that gut symbiotic bacteria in insect pests can degrade pesticides, which improves the resistance level of hosts to such compounds [18, 19].

It has been reported that *Pseudomonas* species could degrade imidacloprid, such as *Pseudomonas putida* [20], *Pseudoxanthomonas indica* CGMCC 6648 [21], *Pseudomonas* sp. 1G, and *Pseudomonas* sp. RPT 52 [22]. In addition, other genera of bacteria have also been found to metabolize imidacloprid, such as *Stenotrophomonas maltophilia* CGMCC 1.1788 [23], *Leifsonia* sp. PC-21[24], *Klebsiella pneumonia* BCH1 [25], *Bacillus alkalinitrilicus* [26], *Mycobacterium* sp. MK6 [27], *Ochrobactrum thiophenivorans*, and *Sphingomonas melonis* [28]. Since the metabolites of imidacloprid showed a relatively low affinity for nAChR, their insecticidal activity was significantly decreased in insects [29]. Notably, many insects harbor symbionts within their gut lumen, body cavity, or cells [30]. A number of insects have developed symbiotic microorganisms that can degrade toxic compounds, and these symbiotic microorganisms could contribute to host resistance against phytotoxins and pesticides [31]. Thus, the degradation of pesticides by insect symbionts could be an important resistance mechanism of insects to pesticides.

Insects can maintain endosymbiotic bacteria in their body [32]. Detoxifying symbioses can enhance the resistance of the host to pesticides [33]. Susceptible *Riptortus pedestris* developed resistance to fenitrothion via the acquisition of gut symbionts of the genus *Burkholderia*, which could degrade fenitrothion [18]*.* The symbionts of *Bacillus cereus* isolated from the gut of the diamondback moth *Plutella xylostella* were identified to degrade indoxacarb with high efficiency, which resulted in the resistance of *P. xylostella* to indoxacarb [34]. The gut symbiont *Citrobacter* sp. of the oriental fruit fly *Bactrocera dorsalis* played a key role in the degradation of trichlorfon, which contributed to the decreased susceptibility of the host to trichlorfon [19]. Moreover, the gut bacteria isolated from the resistant strains of *Spodoptera frugiperda* possessed pesticide-degrading capacity, such as cyhalothrin, deltamethrin, chlorpyrifos-ethyl, spinosad, and lufenuron, which could be degraded efficiently [35].

In our study, we hypothesized that gut symbionts of *A. gossypii* could increase its resistance to imidacloprid by degrading such compounds. 16S rDNA sequencing was performed to evaluate the differential abundance of the gut symbionts at the genus level between the imidacloprid-resistant (IMI-R) and imidacloprid-susceptible (IMI-S) strains of *A. gossypii*. We then isolated and cultivated the gut symbiont *Sphingomonas* to examine its ability to degrade imidacloprid. Both deprivation or supplementation of *Sphingomonas* was conducted in the IMI-R and IMI-S strains, and then the susceptibility of *A. gossypii* to imidacloprid was analyzed by the leaf-dipping method. The prevalence of infection with *Sphingomonas* in nine field populations was detected by quantitative real-time PCR (qPCR). The changes in the susceptibility of field populations to imidacloprid were further investigated after antibiotic treatment. Finally, the metabolic capacity and metabolic pathway of imidacloprid by *Sphingomonas* were determined by high-performance liquid chromatography coupled to tandem mass spectrometry (HPLC–MS).

## Results

### The higher relative abundance of *Sphingomonas *in the imidacloprid-resistant strain

The toxicity of imidacloprid to IMI-S and IMI-R strains of *A. gossypii* was determined (Table 1). Compared to the IMI-S strain with an LC_50_ value of only 3.14 mg/L, the IMI-R strain developed high resistance to imidacloprid with an LC_50_ value greater than 5000 mg/L, and the resistance ratio was more than 1592.

**Table 1:** Toxicity of imidacloprid to IMI-S and IMI-R strains of *Aphis gossypii* after treatment with ampicillin or *Sphingomonas* for seven days

Strains	Treatment	LC_50_ (95% CL) (mg/L)^a^	Resistance ratio (RR)^b^
IMI-S		3.14 (2.19–4.15)	1
IMI-R		> 5000	> 1592
IMI-S	Ampicillin	1.85 (0.88–3.29)	0.59
IMI-R	Ampicillin	1656.71 (1005.60–2860.60)	528
IMI-S	*Sphingomonas*	19.99 (12.41–31.66)	6.37
IMI-R	*Sphingomonas*	> 5000	> 1592

^a^Confidence limits

^b^RR (resistance ratio) = LC_50_ value of the IMI-R/IMI-S (IMI-R/IMI-S strain treated by ampicillin or *Sphingomonas* for 7 days) strain/LC_50_ of the IMI-S strain

To identify which gut symbiotic bacteria in *A. gossypii* might contribute to imidacloprid metabolism, we analyzed the bacterial composition of gut communities in both IMI-S and IMI-R strains by sequencing the V4 variable region of 16S rDNA with high-throughput amplicon sequencing (GenBank accession No. PRJNA929464). As shown in Additional file 1: Table S1, the reads generated from the samples of IMI-R and IMI-S strains were all more than 80,000. The value of Good’s coverage from all the samples was closer to 1, indicating that the sequencing depth basically covered all species in the samples (Table S1). Rarefaction curves were generated to avoid biases in the downstream analyses, which directly reflected that the amount of sequencing data of all the samples was rational (Additional file 2: Fig. S1).

**Fig. 1:**
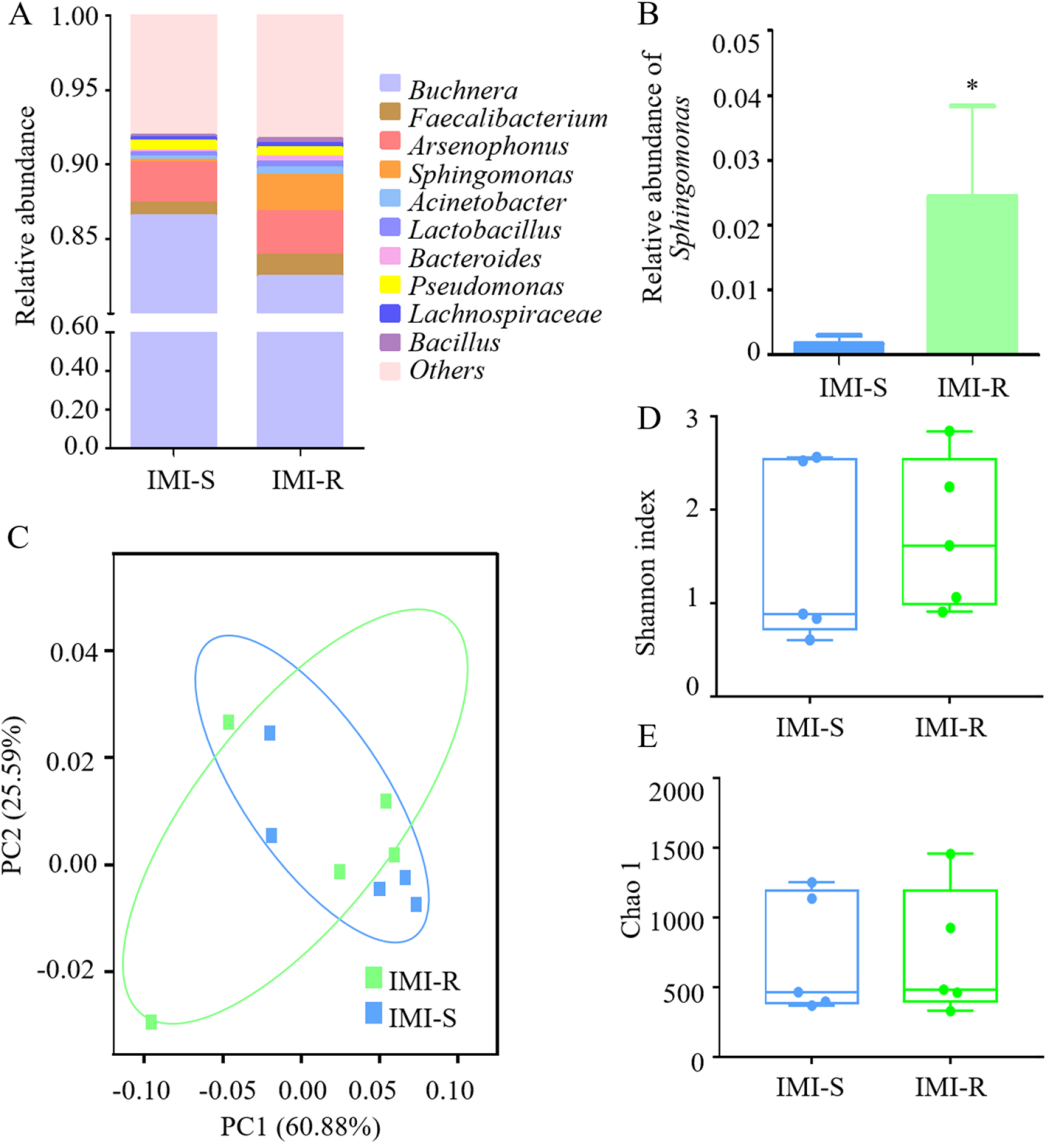
Different symbiotic bacterial communities in the *Aphis gossypii* guts of IMI-S and IMI-R strains. **A** Relative abundances of the top 10 genera in the guts of the IMI-S and IMI-R strains. **B** The relative abundance of *Sphingomonas* in the guts of the IMI-S and IMI-R strains. Asterisks indicate significant differences between strains followed by *t* test, *n* = 5; “*”, *P* < 0.05. **C** The PCoA plot determined by the structure and composition of gut symbionts in the IMI-S and IMI-R strains based on weighted UniFrac analysis. Alpha diversity indices of species for the gut symbiont in the IMI-S and IMI-R strains, Shannon index (**D**) and Chao 1 index (**E**) based on the Wilcoxon test, *n* = 5

At the genus level, the relative abundance was evaluated for all observed genera. The gut microbiota of both IMI-S and IMI-R strains was mainly composed of *Buchnera*, *Faecalibacterium*, *Arsenophonus*, and *Sphingomonas* (Fig. 1A). The relative abundance of *Sphingomonas* was significantly higher in the IMI-R strain than in the IMI-S strain (Fig. 1B). Principal coordinate analysis (PCoA) showed that the IMI-S and IMI-R strains were clustered together, indicating that the structure and composition of gut symbionts in the IMI-R strain were not significantly different from those of the IMI-S strain (Fig. 1C). Shannon index analysis demonstrated that there was no significant difference in the diversity of the gut symbionts between the two strains (Fig. 1D). The Chao1 index (reflecting species richness of bacteria) in the IMI-R strain was also similar to that in the IMI-S strains (Fig. 1E).

### *Sphingomonas* isolation and identification

**Fig. 2:**
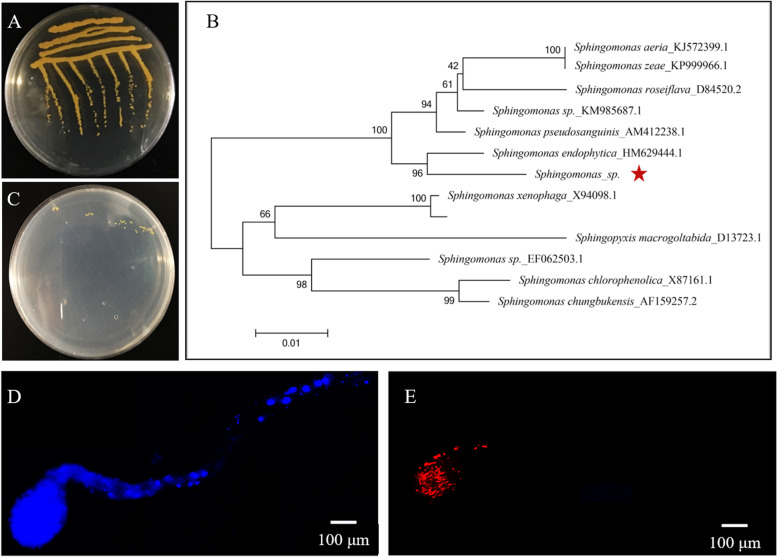
Isolation, identification, and location of *Sphingomonas*. **A** The colony characteristics of *Sphingomonas* on 2xYT agar plates. **B** Phylogenetic relationships of the symbiotic *Sphingomonas* strain. The red star indicates the *Sphingomonas* strain isolated from the gut of the IMI-R strain. **C** The colony characteristics of *Sphingomonas* on mineral media. **D**, **E** The gut organization of *A. gossypii* and localization of *Sphingomonas* in the gut of the IMI-R strain. Red signals indicate *Sphingomonas* symbionts, whereas blue signals show host insect nuclei

Guts of *A. gossypii* were dissected on sterilized ice-cold slides and plated on 2xYT agar plates to isolate *Sphingomonas.* According to the 16S rDNA sequencing results, bacteria were identified as *Sphingomonas* (Fig. 2A). Based on a BLAST search against GenBank, the 16S rDNA sequence exhibited 99% identity with that of *Sphingomonas* (Fig. 2B)*.* The 16S rDNA fragment of the bacteria was 1314 bp, and the sequence was deposited in GenBank (accession number: OQ359156).

### Imidacloprid-degrading bacteria of *Sphingomonas* in the gut of *Aphis gossypii*

Carbon and nitrogen sources are indispensable to bacterial subsistence. *Sphingomonas* could subsist on the mineral media, which used imidacloprid as a sole carbon and nitrogen source (Fig. 2C). According to Fig.S2, the OD_600_ value of *Sphingomonas* was significantly increased after being cultivated in the liquid MM with IMI as the only carbon source and nitrogen source for 2 days. but no significant change of OD_600_ value was observed for *Sphingomonas* cultivated in liquid MM without IMI as the only carbon source and nitrogen source during the 3-day experiment. The above results indicated that carbon and nitrogen from imidacloprid were utilized by *Sphingomonas* to subsist.

### Localization of *Sphingomonas* symbionts in guts

Fluorescence in situ hybridization (FISH) targeting 16S rDNA of *Sphingomonas* symbionts was performed to identify its location in the gut of *A. gossypii.* The fluorescent signal of *Sphingomonas* was consistently localized in the foregut of *A. gossypii* (Fig. 2D, E).

### Deprivation of *Sphingomonas* increased *A. gossypii* susceptibility to imidacloprid

**Fig. 3:**
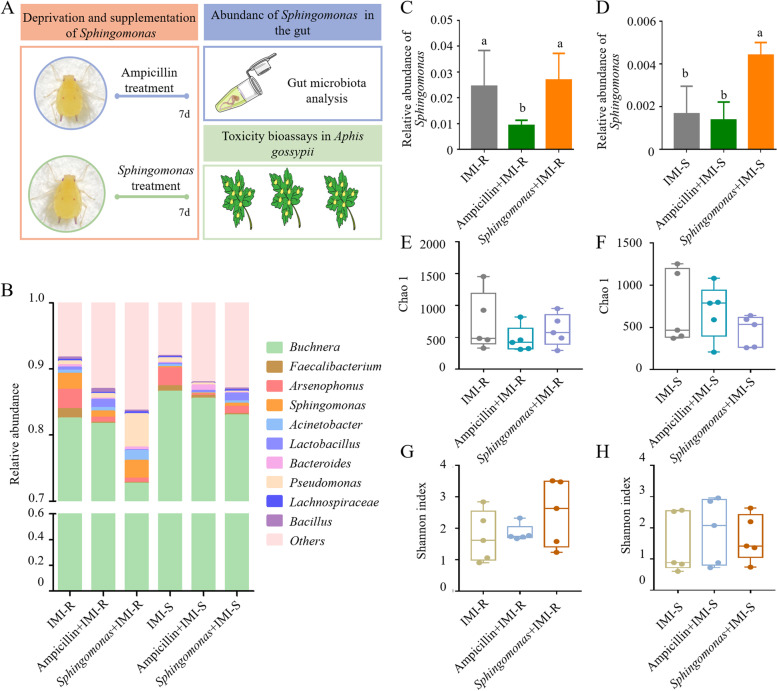
Deprivation and supplementation of *Sphingomonas* in the gut of the IMI-S and IMI-R strains and detection of *Sphingomonas* in nine field populations of *Aphis gossypii* collected in 2019. **A** Experimental design: apterous adult aphids of the IMI-S and IMI-R strains were treated with ampicillin for 7 days to deprive *Sphingomonas* or treated with *Sphingomonas* for 7 days for *Sphingomonas* supplementation*.* Afterward, the guts of apterous aphids from the IMI-S strain or IMI-R strain were dissected for DNA extraction and then for 16S rDNA sequencing. In addition, after treatment with ampicillin or *Sphingomonas* for 7 days, the changes in the susceptibility of the IMI-S and IMI-R strains to imidacloprid were determined by the leaf-dipping method. **B** Histogram showing the relative abundance of the 10 most dominant bacterial genera of the gut symbiont in the IMI-R and IMI-S strains. The abundance of *Sphingomonas* in the gut of the IMI-R and IMI-S strains (**C**, **D**). The different lowercase letters (a, b, c) on the bars indicate significant differences according to one-way ANOVA (*n* = 5, *P* < 0.05, Tukey’s test). The analysis of alpha diversity indices of species for the gut symbiont in the IMI-S and IMI-R strains, Chao 1 index (**E**, **F**) and Shannon index (**G**, **H**) based on the Wilcoxon test, *n* = 5

We examined whether the deprivation or supplementation of *Sphingomonas* affects the susceptibility of IMI-R and IMI-S strains to imidacloprid. Thus, 16S rDNA gene sequencing and toxicity bioassays were performed on both strains after treatment with antibiotics or *Sphingomonas* for seven days (Fig. 3A). The susceptibility of *Sphingomonas* to antibiotics was tested by comparison of the inhibiting zone. The results demonstrated that *Sphingomonas* is highly sensitive to ampicillin (Additional file 3: Fig. S2). Therefore, ampicillin was used to remove *Sphingomonas* from the guts of the IMI-R and IMI-S strains. According to the sequencing result of 16S rDNA (accession number PRJNA929464), the reads generated from the samples of IMI-R and IMI-S strains after being treated with ampicillin or *Sphingomonas* for 7 days were all more than 60,000 (Table S1). The Good’s coverage values suggested that the sequencing was sufficiently deep to cover all species in the samples (Table S1). Rarefaction curves were generated, indicating that the amount of sequencing data was rational in the samples (Additional file 2: Fig. S1).

The top 10 bacterial genera in Fig. 3B revealed that the shifted structure and composition of gut symbionts were observed in the IMI-R strain or IMI-S strain after being treated with ampicillin or *Sphingomonas* for 7 days. It is true that the abundance of *Sphingomonas* from the gut of the IMI-R strain was significantly reduced after treatment with ampicillin for 7 days compared with the control of the IMI-R strain, which was treated with sterile water only (Fig. 3C). After the supplementation of *Sphingomonas* for 7 days in the IMI-R and IMI-S strains, the abundance of *Sphingomonas* from the gut of the IMI-S strain was significantly increased compared with the control treated with sterile water only (Fig. 3D). Measurement of within-sample diversity (α-diversity) by Chao 1 and Shannon index revealed no significant difference in the IMI-R strain or IMI-S strain after being treated with ampicillin or *Sphingomonas* for 7 days (Fig. 3E–H).

Although the LC_50_ value of the IMI-S strain decreased from 3.14 to 1.85 mg/L after treatment with ampicillin for 7 days, the overlapping 95% confidence limits indicated that no significant difference existed between the two LC_50_ values (Table 1). The abundance of *Sphingomonas* in the gut of the IMI-S strain was lower, and it could not be further reduced by antibiotic exposure. Thus, no significant increase in susceptibility was observed in the IMI-S strain. The LC_50_ value of the IMI-R strain decreased from > 5000 to 1656.71 mg/L after ampicillin treatment for 7 days, and the resistance ratio decreased from > 1592 to 528, indicating that the significantly reduced abundance of *Sphingomonas* in the gut could increase the susceptibility of the IMI-R strain to imidacloprid (Table 1).

### Supplementation with *Sphingomonas* decreased *A. gossypii* susceptibility to imidacloprid

The LC_50_ value of the IMI-S strain increased significantly from 3.14 to 19.99 mg/L after supplementation with *Sphingomonas* for 7 days (Table 1). Moreover, the supplementation of *Sphingomonas* increased the resistance of the IMI-S strain to 6.37-fold, indicating that the gut possessed a higher abundance of *Sphingomonas*, dramatically decreasing the susceptibility of *A. gossypii* to imidacloprid. There was no obviously increased abundance of *Sphingomonas* observed in the gut of the IMI-R strain after supplementation. As expected, the LC_50_ value and the resistance ratio for the IMI-R strain showed little change. (Table 1).

### Detection and deprivation of *Sphingomonas* symbionts in the field populations

The results showed that the nine field populations from various provinces in China were all infected with *Sphingomonas* (Additional file 4: Fig. S3). After these field populations were treated with ampicillin for 7 days for the deprivation of *Sphingomonas*, their susceptibilities to imidacloprid were all decreased to different degrees (Table 2). Among these nine field populations, the SXYC population possessed the highest LC_50_ value of 19,830 mg/L with a resistance ratio of 6315 but dramatically decreased to 844 mg/L (RR = 269) after treatment with ampicillin for 7 days. The resistance ratio of the SXYC population showed the greatest decrease compared to other field populations treated with antibiotics. In addition to the SXYC population, the resistance ratios of the XJWS and SDBZ populations also showed a greater decrease after exposure to ampicillin for 7 days, and their resistance ratios decreased from 55 to 18 and 134 to 37, respectively. However, no obvious decrease in the resistance ratio was observed in the remaining six populations after ampicillin treatment. 16S rDNA sequencing was also performed to determine the abundance of *Sphingomonas* in the gut of SXYC, SDBZ, XJSW, and HBHS field populations (accession No. PRJNA929464). The abundance of *Sphingomonas* in the gut of these four populations was also compared with that of the IMI-S and IMI-R strains. As shown in Additional file 5: Fig. S4, the highest abundance of *Sphingomonas* was observed in the IMI-R strain and SXYC field population, and the lowest was found in the gut of the IMI-S strain, XJSW, and HBHS field populations. Furthermore, the abundance of *Sphingomonas* in the gut of SXYC and SDBZ populations was significantly higher than that in the IMI-S strain.

**Table 2:** Toxicity of imidacloprid to field populations of *Aphis gossypii* after treatment with ampicillin for 7 days

Strains	Treatment	LC_50_ (95% CL) (mg/L)^a^	RR^b^
SXYC		19,830.07 (8217.73–280,352.90)	6315
SXYC	Ampicillin	844.08 (295.00–1574.49)	269
XJAKS		14.89 (0.07–77.43)	5
XJAKS	Ampicillin	12.20 (7.20–18.34)	4
SDDY		2391.80 (1263.84–5208.50)	762
SDDY	Ampicillin	2124.98 (1261.05–3653.50)	677
HBHS		2272.55 (1178.86–5531.05)	724
HBHS	Ampicillin	2111.01 (1201.04–3575.02)	672
XJSW		154.40 (90.40–224.070)	49
XJSW	Ampicillin	96.98 (39.092386.33)	31
XJWS		172.71 (34.36–386.33)	55
XJWS	Ampicillin	55.82 (8.11–127.38)	18
HBJZ		180.29 (14.42–400.70)	57
HBJZ	Ampicillin	132.54 (68.97–211.98)	42
SDJN		15.86 (1.40–47.62)	5
SDJN	Ampicillin	13.54 (3.12–27.02)	4
SDBZ		427.03 (21.66–1208.87)	136
SDBZ	Ampicillin	116.61 (44.82–202.40)	37

^a^Confidence limits

^b^RR, resistance ratio = LC_50_ of the field populations/LC_50_ of the IMI-S strain

### Metabolism of imidacloprid by *Sphingomonas*

**Fig. 4:**
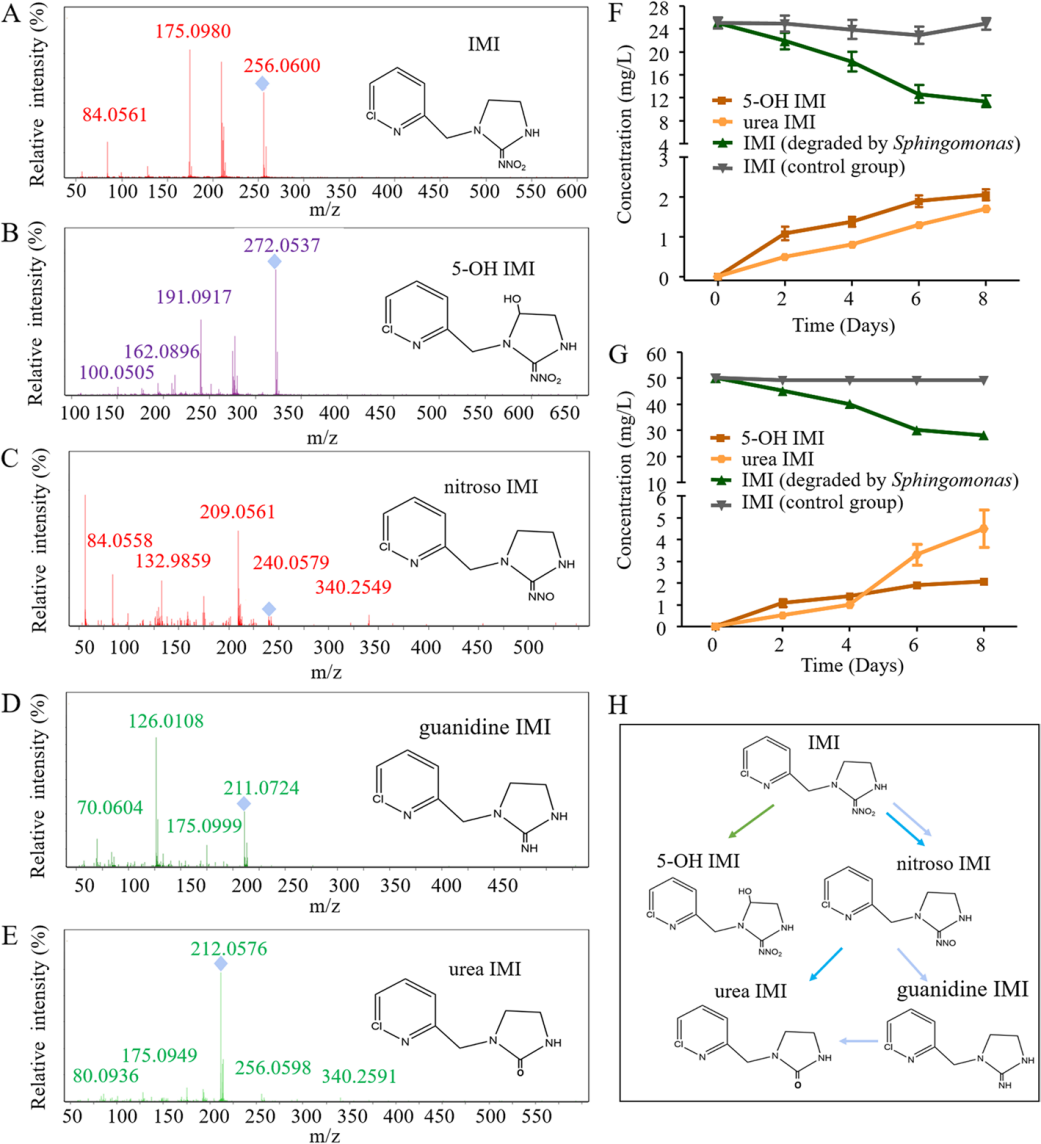
Degradation of imidacloprid by *Sphingomonas*. **A**–**E** HPLC–MS chromatograms and probable structures of metabolites of imidacloprid after incubation with *Sphingomonas* for 8 days. **F**, **G** Changes in imidacloprid and its metabolite concentrations during the 8-day incubation with *Sphingomonas*. The initial concentrations of imidacloprid were 25 ppm (**F**) and 50 ppm (**G**). **H** Proposed pathways of the biodegradation of imidacloprid by* Sphingomonas*

LC/MS was performed to analyze the metabolic pathway of imidacloprid by *Sphingomonas*. Figure 4A–E shows the molecular ion peaks of imidacloprid (IMI), 5-OH IMI, nitroso IMI, guanidine IMI, and urea IMI. The mass charge ratio (MCR) of ion peak A was 256 and was determined to be IMI. The MCR of ion peak B was 272 with one more oxygen atom than A, indicating that B was 5-OH IMI. The MCR of ion peak C was 240 and was one mass of oxygen atom less than that of ion peak A, indicating that it was nitroso IMI. The MCR of ion peak D was 211, which was two oxygen atoms and one nitrogen atom less than that of ion peak A and one hydrogen atom more than that of ion peak A, indicating that ion peak D was guanidine IMI. The MCR of ion peak E was 212, which was one mass of nitrogen atom and one mass hydrogen atom less than that of ion peak D but one more oxygen atom than that of ion peak D, indicating that ion peak E was urea IMI. Thus, the metabolic pathway of imidacloprid by *Sphingomonas* was determined and is shown in Fig. 4H.

The standard curves of IMI, 5-OH IMI, and urea IMI are shown in Additional file 6: Fig. S5. The metabolic ability of *Sphingomonas* to imidacloprid was examined by HPLC, and samples were collected on days 2, 4, 6, and 8 to analyze the concentration of imidacloprid and its metabolites. The concentration of IMI decreased to a minimum, and the concentrations of 5-OH IMI and urea IMI increased to a maximum on day 8. Specifically, the concentration of IMI was decreased to 11 mg/L and 28 mg/L on day 8, achieving maximum metabolic efficiencies of 44% and 56%, respectively, when the initial IMI concentration was 25 mg/L and 50 mg/L (Fig. 4F, G). In the control group, however, no significant degradation of IMI was observed, regardless of whether the initial concentration of IMI was 25 mg/L or 50 mg/L (Fig. 4G).

## Discussion

Symbiont-mediated detoxification of pesticides has attracted increasing attention. Generally, toxin-degrading symbiotic bacteria defend hosts against pesticides in the environment [33]. In this study, the genus *Sphingomonas* isolated from the gut of the IMI-R strain can utilize imidacloprid as a sole carbon source and nitrogen source. During degradation, the *Sphingomonas* symbiont was identified to metabolize imidacloprid via hydroxylation and nitro-reduction pathways with high efficiency. This further provided evidence that *Sphingomonas* could enhance *A. gossypii* resistance to imidacloprid.

Notably, obligate symbionts usually provide key nutrients to compensate for their host’s unbalanced diets [32]. For example, *Buchnera* could provide aphids with the essential amino acids and vitamins missing in plant phloem sap [36], and *Wigglesworthia* could synthesize vitamin B to contribute to tsetse host fitness [37]. From Fig. 1A, the highest abundance of gut symbionts was *Buchnera*, and it was the obligate symbiont in *A. gossypii*. The main role of facultative mutualists is to confer fitness benefits upon hosts, which extend the lifespan and stimulate the fecundity of their carriers [38]. In addition, facultative symbionts can protect their host against parasitoids [39], pathogens [40], and pesticides [41]. Here, we demonstrated that the gut symbiont *Sphingomonas* plays a key role in the degradation of imidacloprid. Thus, it was supposed to act as a facultative symbiont in *A. gossypii*.

*Sphingomonas* was identified as a new genus in 1990 due to its outer membrane component of sphingosine [42]. In 2001, *Sphingomonas* was divided into four new genera, *Sphingomonas*, *Sphingobium*, *Novosphingobium*, and *Sphingopyxis* [43]. *Sphingomonas* is characterized by its degradation of various organic pollutants, such as biphenyl, naphthalene, phenanthrene, dioxin compounds, carbazole, chlorophenol, and various pesticides [44]. It has been reported that *Sphingomonas* sp. DC-6, isolated from soil, can hydrolyze the organophosphorus pesticide dimethoate into dimethoate carboxylic acid and methylamine [45]. *Sphingomonas* sp. CDS-1 could degrade carbamate insecticide carbofuran into carbofuran phenol in soil [46]. Symbiont *Sphingomonas* sp. HJY from chives, in which chlorpyrifos was applied for a long time, was reported to effectively degrade chlorpyrifos [47]. Thus, *Sphingomonas* in imidacloprid*-*resistant *A. gossypii* may have the potential to degrade imidacloprid.

The results of 16S rDNA sequencing demonstrated that the richness and diversity of the gut community showed no significant difference between the IMI-R and IMI-S strains of *Aphis gossypii*. However, the abundance of *Sphingomonas* in the IMI-R strain was significantly higher than that in the IMI-S strain. Indeed, the titer of individual symbiont species could be regulated by insects to respond to pesticide pressures [48]. Furthermore, we found that imidacloprid could be used as a carbon and nitrogen source by *Sphingomonas* (Fig. 2C, Additional file 7: Fig. S6), indicating the potential metabolic capacity of *Sphingomonas* to imidacloprid. Then, the ability of *Sphingomonas* to metabolize imidacloprid was analyzed by HPLC, and high metabolic efficiency (56%) of imidacloprid was observed. Both decreased imidacloprid concentrations and increased concentrations of its two metabolites (5-OH IMI and urea IMI) were also recorded, which provided solid evidence for the involvement of *Sphingomonas* in imidacloprid resistance. It has been reported that the resistant strain of *R. pedestris* possessed a higher abundance of the gut symbiont *Burkholderia*, which could degrade fenitrothion with high efficiency, and susceptible *R. pedestris* developed resistance to fenitrothion via the acquisition of *Burkholderia* [18]*.* Supplementation of *Citrobacter* from the gut of *B. dorsalis*, which was proven to degrade trichlorfon with high efficiency, could result in decreased susceptibility of *B. dorsalis* to trichlorfon [19]. In our study, after supplementation of *Sphingomonas*, the abundance of *Sphingomonas* in the gut of IMI-S strain significantly increased, and the susceptibility to imidacloprid was significantly decreased as expected. These results suggested that the acquisition of insecticides degrading symbionts could increase insecticide resistance in insects.

However, supplementation with *Sphingomonas* had no effect on the abundance of *Sphingomonas* in the gut of the IMI-R strain and its susceptibility to imidacloprid. The possible reason is that the link between detoxifying symbionts infection and insecticide resistance could be a product of physiological tradeoffs [49]. That is, the increase of *Sphingomonas* abundance may play a role in insecticide resistance to some extent, but the abundance will not increase unlimitedly, which may impair the physiological function of the gut. Our results demonstrated that though SXYC population showed almost four times higher resistance to IMI than the IMI-R strain, the *Sphingomonas* abundance is similar in the guts of both SXYC and IMI-R, which also supported our hypothesis that the abundance of *Sphingomonas* in the gut of *A. gossypii* will not continuous increase because of the physiological tradeoffs.

The resistance level of pests to insecticides could be inhibited by antibiotic treatment [50, 51]. Moreover, pests become more susceptibility to toxins after deprivation of detoxifying microorganisms from the gut [19, 52]. Here, we demonstrated that the susceptibility of the IMI-R strain to imidacloprid was significantly increased after suppressing the abundance of *Sphingomonas* in the gut by antibiotic treatment. Besides, *Sphingomonas* infection was prevalent in the field populations of *A. gossypii*, of which the SXYC, XJWS, and SDBZ populations showed a decreased susceptibility to imidacloprid after antibiotic treatment. However, treatment with ampicillin did not increase the susceptibility to imidacloprid in the remaining six populations. The possible reason is that these populations possess a lower abundance of *Sphingomonas*, and its contribution to imidacloprid resistance is negligible. The *Sphingomonas* abundance data shown in Additional file 5: Fig. S4 partly supported our speculation, that is, among all four tested field populations, the SXYC and SDBZ populations had higher *Sphingomonas* abundance and ampicillin treatment could increase their susceptibility, which is similar to that in the IMI-R strain, while the abundance of *Sphingomonas* in the XJSW and HBHS populations was as low as that in the IMI-S strain, and ampicillin treatment did not affect their susceptibility to imidacloprid. In addition, the origin of *Sphingomonas* in the *A. gossypii* gut may be acquired from the soil because both *Burkholderia* and *Sphingomonas* could be detected in the soil of cotton fields [53], and the gut symbiont *Burkholderia* in a stinkbug has been proven to be acquired by nymphs from the environment every generation [54].

The metabolites of imidacloprid by *Sphingomonas* were further determined by HPLC–MS. The main transformation of IMI occurred via the hydroxylation and nitro-reduction pathways. In the hydroxylation pathway, IMI is metabolized to 5-hydroxy IMI by hydroxylase, and 5-hydroxy IMI is further dehydrated to olefin IMI by dehydratase [55]. Olefin IMI is prone to degradation and eventually decomposes into CO_2_ due to its unsaturated double bonds [56]. In the nitro-reduction pathway, the metabolites of IMI were nitroso IMI, guanidine IMI, and urea IMI under the action of aldehyde oxidase (AOX) [57]. Cytochrome P450s (P450s) and AOX are known to be involved in IMI hydroxylase and nitroreductase, respectively [58]. The metabolites of IMI by *Sphingomonas* were 5-OH IMI, nitroso IMI, guanidine IMI, and urea IMI. We confirmed that IMI was metabolized by *Sphingomonas* via nitroreduction and/or hydroxylation pathways. Metabolite toxicity studies have revealed that the guanidine IMI and urea IMI products formed via the nitro-reduction pathway did not possess any insecticidal activity [59]. It can be concluded that IMI metabolism by *Sphingomonas* via the nitro-reduction pathway was a detoxification process. The results further revealed the mechanisms by which *Sphingomonas* enhances the resistance of *A. gossypii* to imidacloprid. A previous study showed that IMI was metabolized mainly through the hydroxylation pathway in mammals, in which IMI was metabolized to 5-hydroxy IMI and 4,5-dihydroxy IMI by P450s and then converted to olefin IMI after dehydration [60]. Another common metabolic pathway of IMI in mammals is the oxidative cleavage of the methylene group between the pyridine ring and the imidazole ring to generate 6-chloropicolinol, which is further oxidized to 6-chloronicotinic acid [61]. In soil, IMI is metabolized by microbes through the hydroxylation pathway and nitro reduction pathway [62]. Olefin and urea metabolites of IMI were detected in soil samples collected from West Bengal, India [63], and olefin IMI and guanidine IMI were detected in soil collected from Nanjing, China [64]. Our findings showed that the metabolic pathway of IMI by *Sphingomonas* was the same as that of IMI by soil microbes.

## Conclusion

It has been reported that the enhanced detoxifying enzyme activity and target mutations play important roles in imidacloprid resistance in insect pests. Here, we further demonstrated that as a ubiquitous genus in the gut of *A. gossypii*, *Sphingomonas* could enhance the resistance of *A. gossypii* by metabolizing IMI directly. The high metabolic efficiency of *Sphingomonas* to IMI indicated that it may play an important role in defending *A. gossypii* against imidacloprid. These findings enriched our knowledge of mechanisms of insecticide resistance and provided new symbiont-based strategies for control of insecticide-resistant insect pests with high *Sphingomonas* abundance.

## Methods

### *Aphis gossypii *strains

The cotton aphid *Aphis gossypii* Glover was used in this study. The aphid population collected in 2008 from cotton fields in Changchun City of Jilin Province, China, was susceptible to imidacloprid, which was named the imidacloprid-susceptible (IMI-S) strain and maintained in our laboratory without exposure to any insecticides since collection [7]. The imidacloprid-resistant (IMI-R) strain was selected from a field population originally collected from Yuncheng of Shanxi Province by 1000 mg/L imidacloprid for ten generations using the leaf-dipping method. For the leaf-dipping method, approximately 5000 apterous adults were carefully transferred onto cotton leaves and then dipped in 1000 mg/L imidacloprid. In addition, nine field populations of *A. gossypii* collected in August 2020 were also used in this study, including Shanxi Yuncheng (SXYC), Xinjiang Aksu (XJAKS), Shandong Dongying (SDDY), Hebei Hengshui (HBHS), Xinjiang Shawan (XJSW), Xinjiang Wusu (XJWS), Hubei Jingzhou (HBJZ), Shandong Jinan (SDJN), and Shandong Binzhou (SDBZ) populations. Their collection information is listed in Additional file 1: Table S1. All *A. gossypii* strains and populations were reared on cotton seedlings, *Gossypium hirsutum (L.),* under controlled conditions of 20–23 °C, 60% relative humidity, and a photoperiod of 16:8 h (light:dark).

### Toxicity bioassays

The toxicity of imidacloprid to *A. gossypii* was determined using a leaf-dipping method [65] with slight modifications [66]. Stocks of imidacloprid (95.3%) were obtained from DuPont (USA), prepared in acetone and adjusted to final concentrations by serial dilution with distilled water containing 0.05% (v/v) Triton X-100 for the bioassays. The 20-mm diameter cotton leaf discs were dipped in the desired concentration of insecticide or in 0.05% (v/v) Triton X-100 water for 15 s as a control. The treated leaf discs were allowed to air dry and then placed upside down onto the agar beds in 12-well cell culture plates. Apterous adult aphids were carefully transferred onto the discs and covered with Chinese art paper to prevent escape. Bioassays were maintained in the laboratory at 20–23 °C with a photoperiod of 16:8 h (light: dark). The treatment for each concentration was performed with three replicates, and at least 30 aphids were used for each replicate. Mortality was assessed at 48 h after treatment. The LC_50_ values were calculated by probit analysis using POLO Plus 2.0 statistical software (LeOra Software Inc., Berkeley, CA).

### Extraction of DNA from guts of the IMI-S and IMI-R strains and field populations

The DNA of the gut was extracted from IMI-S and IMI-R strains and field populations following previous research [19]. Apterous adults were selected and soaked in 75% ethanol for 90 s to remove surface bacteria. The entire guts were dissected from soaked aphids under a stereomicroscope and were directly transferred into centrifuge tubes containing DNA extraction buffer. For each sample, 90 aphids were dissected, and five independent biological replicates were conducted. Gut DNA was extracted using a DNA extraction kit (Qingke, Beijing, China) following the manufacturer’s instructions, and the DNA was used for gut symbiont high-throughput sequencing analysis.

### V4 amplicon sequencing of the 16S rDNA genes and data analysis

The bacteria were profiled by sequencing the V4 region of the 16S rDNA gene. Primers 505F (5′-CCTAYGGGRBGCASCAG-3′) and 806R (5′-GGACTACN NGGGTATCTAA-3′) were employed for the PCR. All PCRs were carried out with Phusion® High-Fidelity PCR Master Mix (New England Biolabs), and 400–450 bp were chosen for further experiments. Sequencing libraries were generated using TruSeq DNA PCR-Free Sample Preparation Kits (Illumina, USA) following the manufacturer’s recommendations. Index codes were added. The library quality was assessed on the Qubit@ 2.0 Fluorometer (Thermo Scientific) and Agilent Bioanalyzer 2100 system. Finally, the library was sequenced on an Illumina HiSeq 2500 platform, and 250-bp paired-end reads were generated.

Paired-end reads were merged using FLASH (V1.2.7, http://ccb.jhu.edu/software/FLASH/), and the splicing sequences were called raw tags. After quality filtering of the raw tags, the effective tags were obtained. For species annotation, sequences with 97% similarity were assigned to the same OTUs, and the representative sequence for each OTU was screened for further annotation. For each representative sequence, the GreenGene Database (http://greengenes.lbl.gov/cgi-bin/nph-index.cgi) was used based on the RDP classifier (Version 2.2, http://sourceforge.net/projects/rdp-classifier/) algorithm to annotate taxonomic information.

### *Sphingomonas* isolation and identification

Apterous adult aphids were collected and soaked in 75% ethanol for 90 s, followed by three rinses with sterile water to remove surface bacteria. The guts of *A. gossypii* were dissected and homogenized with 200 μL of sterile water. After five repetitions of tenfold gradient dilutions, all six samples were plated on 2xYT agar plates (0.5% peptone, 0.3% yeast extract, 0.5% NaCl, 1.5% agar, pH 6.8) and cultivated at 27 °C for 48 h. Colonies with distinct morphology were selected for purification on 2xYT medium at least three times and then used for subculturing.

Isolated colonies were transferred to centrifuge tubes containing 5 ml of 2xYT liquid medium, shaken, and incubated at 27 °C for 48 h for DNA extraction. Members of the *Sphingomonas* genus are gram-negative [67]. The bacterial DNA was extracted with Ezup Column Bacteria Genomic DNA Purification Kits (Shenggong, Shanghai, China) following the protocol for gram-negative bacteria. The DNA was used for the amplification of the complete 16S rDNA genes, and primers 27F (5′-GTTTG ATCCTGGCTCAG-3′) and 1492R (5′-GGTTACCTTGTTACGACTT-3′) were employed. PCR amplification was performed using 2xTaq PCR MasterMix (Aidlab, Beijing China), with an initial denaturation at 95 °C for 4 min followed by 30 cycles of 30 s at 95 °C, 30 s at 57 °C, 2 min at 72 °C, and a final extension at 72 °C for 10 min. The sequences of 16S rDNA were subjected to a BLAST search against the NCBI database for sequence homology analysis and bacterial species identification. Liquid cultures of *Sphingomonas* were stored in 25% glycerol solution at − 80 °C.

### Fluorescence in situ hybridization

The location of *Sphingomonas* in the gut of *A. gossypii* was determined by fluorescence in situ hybridization (FISH). *Sphingomonas* symbionts were visualized with a probe SPH120 targeting a specific region of the 16S rDNA (5′-GGGCAGATTCCCACGCGT-3′) [68]. Oligonucleotides were labeled with the fluorophore Texas red by the manufacturer (EXON Biological Technology, Guangzhou, China). Ten gut samples were collected directly into Carnoy’s solution (ethanol-chloroform–acetic acid, 6:3:1) for fixation. After fixation, the samples were bleached in a 6% H_2_O_2_ ethanol solution and then hybridized overnight at 42 °C in hybridization buffer (20 mM Tris–HCl pH 8.0, 0.9 M NaCl, 0.01% SDS, and 35% (wt/vol) formamide) containing 40 nM oligonucleotide probe; the samples were placed in a humid and dark chamber. To remove nonspecific probe binding, the samples were washed in washing buffer (20 mM Tris/HCl pH 7.5, 70 mM NaCl, 0.01% SDS, 5 mM EDTA) for 30 min at room temperature. In addition, 4′,6-diamidino-2-phenylindole (DAPI) was supplied to counter-stain eukaryotic and bacterial DNA for 10 min, which contained an anti-fluorescence quencher. Subsequently, the samples were observed under an inverted fluorescence microscope (Nikon, USA).

### Identification of the imidacloprid-degrading function of *Sphingomonas*

To preliminarily identify the ability of *Sphingomonas* to degrade imidacloprid, isolated colonies were lined on mineral media (MM) (9.5 mM KH_2_PO_4_, 4.8 mM MgSO_4_, 0.1 mM CaCl_2_, 0.8 mM Na_2_HPO_4_, and 20 g/L bacto agar), and 5 mM imidacloprid was added and used as the sole carbon and nitrogen source. The growth of bacteria was monitored daily to confirm whether *Sphingomonas* could subsist on the mineral media. In addition, the changes in OD value of *Sphingomonas* cultivated in the liquid mineral media (MM) (9.5 mM KH_2_PO4, 4.8 mM MgSO_4_, 0.1 mM CaCl_2_, 0.8 mM Na_2_HPO_4_) with IMI as the only carbon source and nitrogen source was observed, and the liquid MM without carbon and nitrogen source was used as the control. The inoculum of *Sphingomonas* was centrifuged at 8000 rpm and then suspended into 50-mL liquid MM with 25 ppm IMI as the only carbon source and adjusted the initial OD_600_ 0.8. The inoculated media were incubated at 28 °C, 180 rpm for 3 days. Take out 1-mL cultures every 24 h and use ultraviolet spectrophotometer (Spectra Max; Molecular Devices) to measure the OD value of *Sphingomonas* at 600 nm. The MM (50 mL) without carbon source was used as the control. Three replicates were prepared for each treatment.

### Antibiotic sensitivity testing of *Sphingomonas*

The sensitivity of *Sphingomonas* to antibiotics was investigated using the method of inhibiting zone. Ten microliters of the inoculated *Sphingomonas* with an OD600 of 0.8 was mixed with 20 mL 2xYT agar plates, and 60 μl of antibiotics with concentrations of 0.5 mg/L, 1 mg/L, 2 mg/L, and 4 mg/L were added to a hole of 0.3-cm diameter in the center of the agar plate. After cultivation at 27 °C for 48 h, the diameter of the inhibiting zones was measured. The types of antibiotics are shown in Additional file 5: Fig. S4.

### Antibiotic treatment and *Sphingomonas* inoculation

According to the results of antibiotic sensitivity testing, a 10 mg/ml ampicillin solution was selected to remove *Sphingomonas.* Here, 100 apterous adult aphids were carefully transferred onto 80-mm diameter cotton leaf discs and then treated with 100 μl ampicillin by nanospraying for 7 days, and *A. gossypii* in the control group were treated with ampicillin-free water. Each antibiotic or ampicillin-free water treatment was performed with ten replicates. Then, the guts of apterous adult aphids from both the IMI-S and IMI-R strains were dissected for 16S rDNA sequencing. The changes in toxicity of imidacloprid to IMI-S and IMI-R strains after being treated with antibiotics were examined using the leaf-dipping method, and the biometric data were analyzed to determine the changes in imidacloprid susceptibility compared to the control.

For the *Sphingomonas* inoculation experiments, isolated *Sphingomonas* were propagated in 2xYT liquid medium at 27 °C and 200 rpm until an OD600 of 0.8 was reached. One hundred apterous adult aphids were carefully transferred onto 80-mm diameter cotton leaf discs and then treated with 100 μl *Sphingomonas* inoculum by nanospraying for 7 days, and *A. gossypii* in the control group were treated with sterilized water. Each *Sphingomonas* or sterilized water treatment was performed with ten replicates. The guts of apterous adult aphids were dissected for 16S rDNA sequencing. After being treated with *Sphingomonas* for 7 days, the susceptibility of the IMI-S and IMI-R strains to imidacloprid was determined by the leaf-dipping method.

### Antibiotic treatment in field populations

Nine field populations from different provinces of China were collected in 2019, and the collection information is listed in Additional file 8: Table S2. The infection of *Sphingomonas* was detected by the cycle threshold (Ct) value for each population according to the result of amplification of the 16S rDNA gene by quantitative real-time PCR (qPCR). For qPCR detection, we isolated genomic DNA from the nine populations of apterous adults using a DNA extraction kit (Qingke, Beijing, China), and qPCR was performed on an Applied Biosystems 7500 Real-Time PCR system (Applied Biosystems, Foster City, CA, USA) using SYBR Premix Ex Taq (Tli RNaseH Plus) (Takara Biotechnology, Dalian, China). The qPCRs were prepared as follows: 1 μg of DNA as template, 10 μL of SYBR Green mix, 0.4 μL of ROX Reference Dye II, 0.4 μL of each primer and DEPC-treated water to a final volume of 20 μl. The specific primers of the 16S rDNA gene to amplify *Sphingomonas* were SP-190 (5′-CGGACCAAAGATTTATCG-3′) and SP-853 (5′-CCAATCACCAAGTGACCCGGA-3′) [67]. The qPCR conditions were 95 °C for 30 s, followed by 40 cycles of 95 °C for 5 s and 60 °C for 34 s. After amplification, one dissociation step cycle of 95 °C for 15 s, 60 °C for 1 min and 95 °C for 30 s, and 60 °C for 15 s was performed to ensure the specificity of the amplified product. The experiment was conducted with three technical replications and three independent biological replicates.

Nine field populations from different provinces of China in 2019 were treated with ampicillin to examine the changes in susceptibility to imidacloprid. A 10 mg/ml ampicillin solution was sprayed onto the infected populations evenly for 7 days. Afterward, the leaf-dipping method was performed to determine the changes in susceptibility to imidacloprid in these nine field populations of *A. gossypii*.

### Degradation of imidacloprid by *Sphingomonas*

Degradation tests were performed using imidacloprid as a sole source of carbon in Erlenmeyer flasks containing 50 mL liquid MM supplemented with 25 mg/L or 50 mg/L imidacloprid. To measure the degradation ability of *Sphingomonas*, 10-mL inoculum with an OD600 of 0.8 was centrifuged at 8000 rpm, and then the collected bacteria were added to MM. The MM without *Sphingomonas* was used as the control. Three replicates were prepared for each treatment. The flasks were incubated at 28 °C and 180 rpm for 8 days. Every 2 days, 1 mL of the cultures was filtered through a 45-μm bacterial membrane filter to detect the concentration of imidacloprid and its metabolites. The concentration of imidacloprid and its metabolites was determined at 269 nm by HPLC (1260 series; Agilent) on a Zorbax SB-Aq C^18^ column (5 μm, 4.6 mm 250 mm; Agilent). The mobile phase consisted of a mixture of 75% ultrapure water: 25% acetonitrile at a flow rate of 1.0 mL/min; the injection volume was 10 μL. To determine the degradation ability of *Sphingomonas*, the concentrations of imidacloprid and its metabolites were calculated by standard curves of imidacloprid, urea-imidacloprid, and 5-OH imidacloprid. Imidacloprid (DuPont, USA), urea imidacloprid (Standards, Shanghai, China), and 5-OH imidacloprid (Xiyuan, Shanghai, China) were prepared in acetonitrile and adjusted to final concentrations by serial dilution with acetonitrile. The standard curve was obtained according to the peak area corresponding to the concentration of compounds.

The metabolic pathways of imidacloprid by *Sphingomonas* were determined by an Agilent 1260/6520 liquid chromatography-mass spectrometer. MS analysis was performed in electrospray ionization (ESI) mode with an Agilent 1290 LC–MSD to analyze and identify imidacloprid and its metabolites. LC conditions: the chromatographic column and mobile phase were the same as those of HPLC; otherwise, the flow rate was 0.6 mL/min. A diode array detector (DAD) was utilized. In the MS analysis, the metabolites were separated and ionized by electrospray ionization to obtain a positive polarity. Characteristic fragment ions were identified by second-order MS and compared to those generated with authentic or structural analog standards.

## Supplementary Information

**Additional file 1: Table S1.** Total reads and good coverage in different samples. 

**Additional file 2: Fig. S1.** Rarefaction curves obtained from all samples.

**Additional file 3: Fig. S2.** The susceptibility of Sphingomonas to different antibiotics. (A) The inhibition zone of different antibiotics against Sphingomonas (a-f): sodium sulfate, gentamicin, chloramphenicol, tetracycline, amoxicillin, and ampicillin. (B) The line chart of different antibiotics to Sphingomonas.

**Additional file 4: Fig. S3.** Detection of Sphingomonas in nine field populations in 2019. Bars represent the mean ± SE (*P* < 0.05, Tukey’s test).

**Additional file 5: Fig. S4.** The abundance of Sphingomonas in the gut of the IMI-S and IMI-R strains and SXYC, SDBZ, XJSW and HBHS field populations. The bars with lowercase letters (a, b, c) are significantly different according to one-way ANOVA, followed by Tukey's multiple comparison test (*P*< 0.05).

**Additional file 6: Fig. S5.** The stand curve of IMI (A), urea IMI (B) and 5-OH IMI (C).

**Additional file 7: Fig. S6.** The changes in OD600 value of Sphingomonas in the control group (A) and IMI group (B) during the 3-day cultivation. The bars with different lowercase letters (a, b, c) are significantly different (one-way ANOVA followed by Tukey's multiple comparison, *P*< 0.05).

**Additional file 8: Table S2.** Collecting information of Aphis gossypii field populations.

## Data Availability

16 s amplicon sequencing data are available from NCBI with accession No. PRJNA929464 [69]. The 16S rDNA fragment of *Sphingomonas* was deposited in GenBank (accession No. OQ359156)[70].
