# Supplementary material for: The gut symbiont Sphingomonas mediates imidacloprid resistance in the important agricultural insect pest Aphis gossypii Glover
Source: BMC Biol. 2023 Apr 17;21:86. doi: 10.1186/s12915-023-01586-2 (PMC10111731; doi:10.1186/s12915-023-01586-2)
Supplement: Supplementary file 1 — Additional file 1: Table S1. Total reads and good coverage in different samples. [file 12915_2023_1586_MOESM1_ESM.docx]

Table S1 Total reads and goods coverage in different samples.

| Sample_name | Total reads | Goods coverage |
| --- | --- | --- |
| IMI-R1 | 91,296 | 0.996 |
| IMI-R2 | 85,021 | 0.999 |
| IMI-R3 | 93,650 | 0.998 |
| IMI-R4 | 99,395 | 0.998 |
| IMI-R5 | 90,992 | 0.997 |
| IMI-S1 | 93,219 | 0.999 |
| IMI-S2 | 93,949 | 0.996 |
| IMI-S3 | 81,494 | 0.996 |
| IMI-S4 | 91,590 | 0.998 |
| IMI-S5 | 82,059 | 0.999 |
| IMI-R+ampicillin1 | 60385 | 0.998 |
| IMI-R+ampicillin2 | 65301 | 0.998 |
| IMI-R+ampicillin3 | 63654 | 0.996 |
| IMI-R+ampicillin4 | 69680 | 0.997 |
| IMI-R+ampicillin5 | 83878 | 0.999 |
| IMI-R*+Sphingomonas1* | 91352 | 0.999 |
| IMI-R*+Sphingomonas2* | 84286 | 0.998 |
| IMI-R*+Sphingomonas3* | 90528 | 0.999 |
| IMI-R*+Sphingomonas4* | 83548 | 0.998 |
| IMI-R*+Sphingomonas5* | 68186 | 0.997 |
| IMI-S+ampicillin1 | 82867 | 0.997 |
| IMI-S+ampicillin2 | 81648 | 0.999 |
| IMI-S+ampicillin3 | 84258 | 0.999 |
| IMI-S+ampicillin4 | 86974 | 0.997 |
| IMI-S+ampicillin5 | 68113 | 0.998 |
| IMI-S*+Sphingomonas1* | 89529 | 0.997 |
| IMI-S*+Sphingomonas2* | 78903 | 0.997 |
| IMI-S*+Sphingomonas3* | 89064 | 0.995 |
| IMI-S*+Sphingomonas4* | 79436 | 0.999 |
| IMI-S*+Sphingomonas5* | 82002 | 0.997 |
