# Supplementary figures and images for: The gut symbiont Sphingomonas mediates imidacloprid resistance in the important agricultural insect pest Aphis gossypii Glover
Source: BMC Biol. 2023 Apr 17;21:86. doi: 10.1186/s12915-023-01586-2 (PMC10111731; doi:10.1186/s12915-023-01586-2)

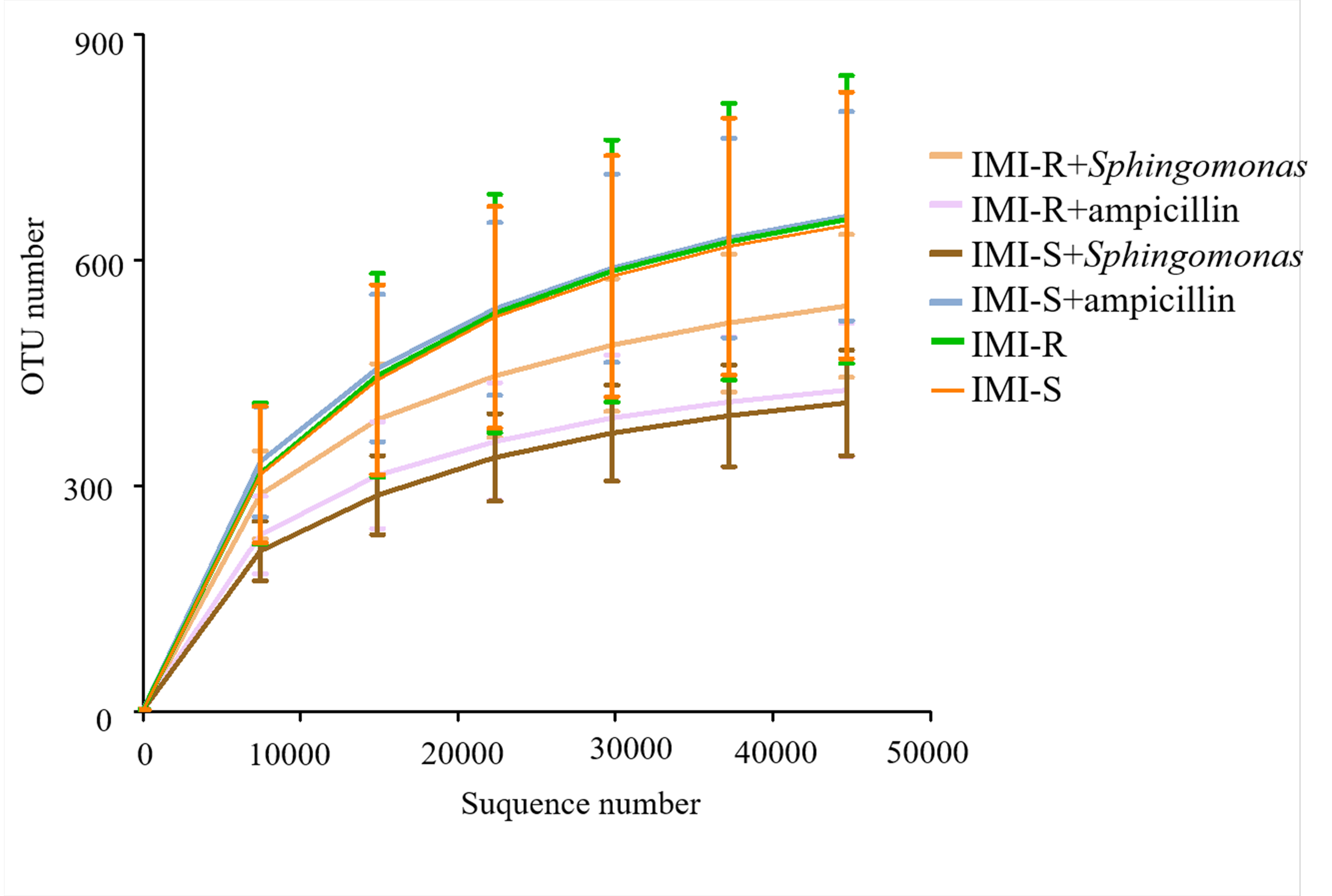

Supplement: Supplementary file 2 — Additional file 2: Fig. S1. Rarefaction curves obtained from all samples. [file 12915_2023_1586_MOESM2_ESM.tif]

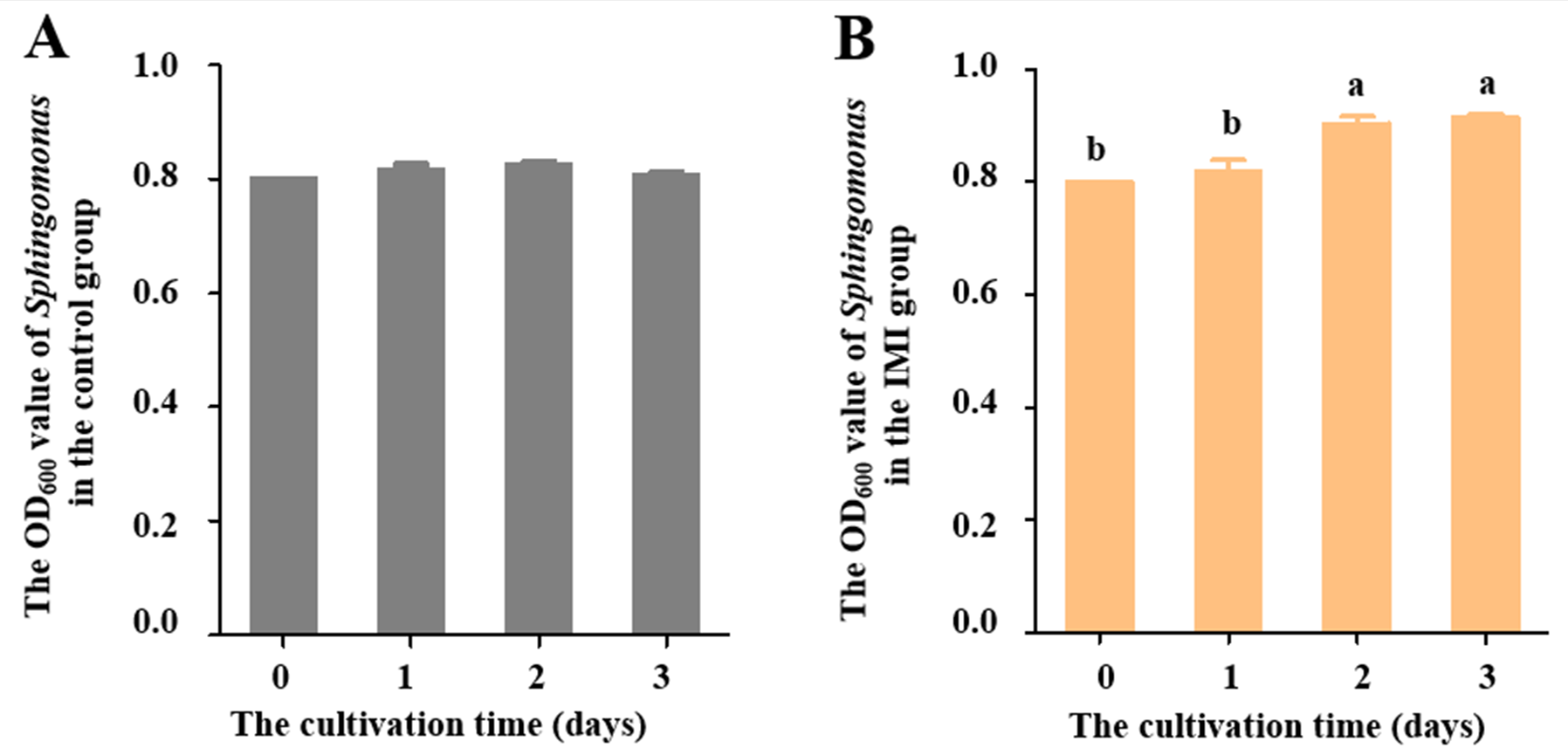

Supplement: Supplementary file 3 — Additional file 3: Fig. S2. The susceptibility of Sphingomonas to different antibiotics. (A) The inhibition zone of different antibiotics against Sphingomonas (a-f): sodium sulfate, gentamicin, chloramphenicol, tetracycline, amoxicillin, and ampicillin. (B) The line chart of different antibiotics to Sphingomonas. [file 12915_2023_1586_MOESM3_ESM.tif]

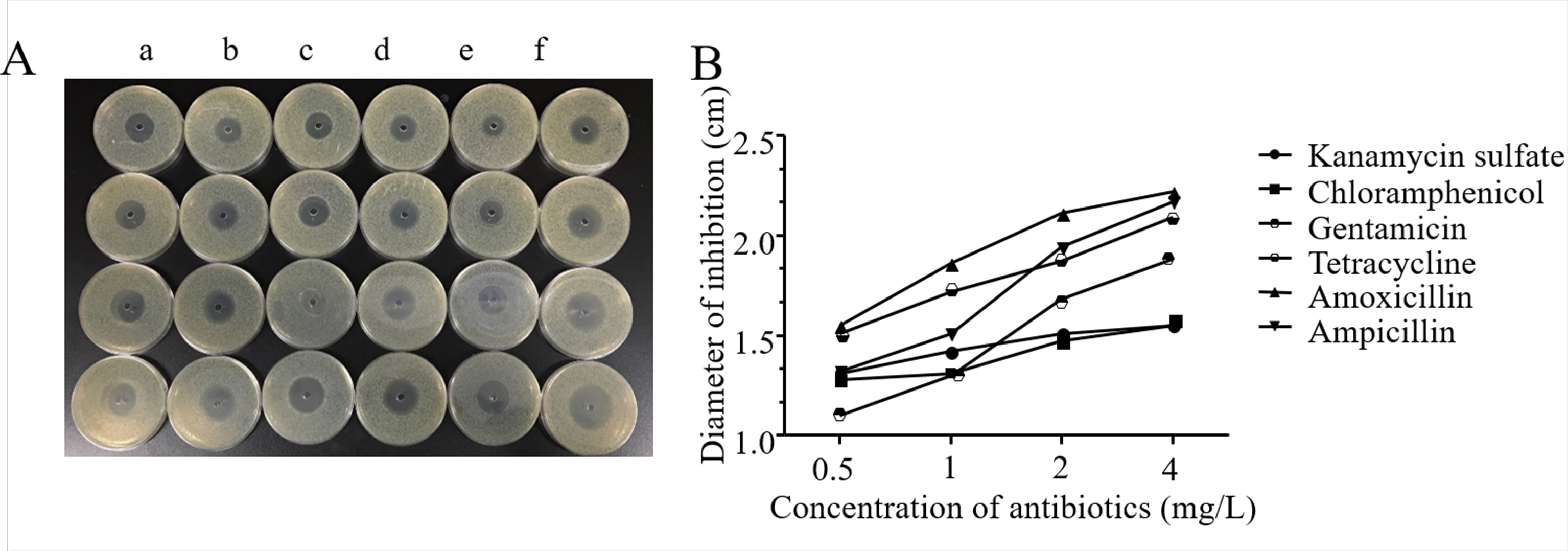

Supplement: Supplementary file 4 — Additional file 4: Fig. S3. Detection of Sphingomonas in nine field populations in 2019. Bars represent the mean ± SE (P < 0.05, Tukey’s test). [file 12915_2023_1586_MOESM4_ESM.tif]

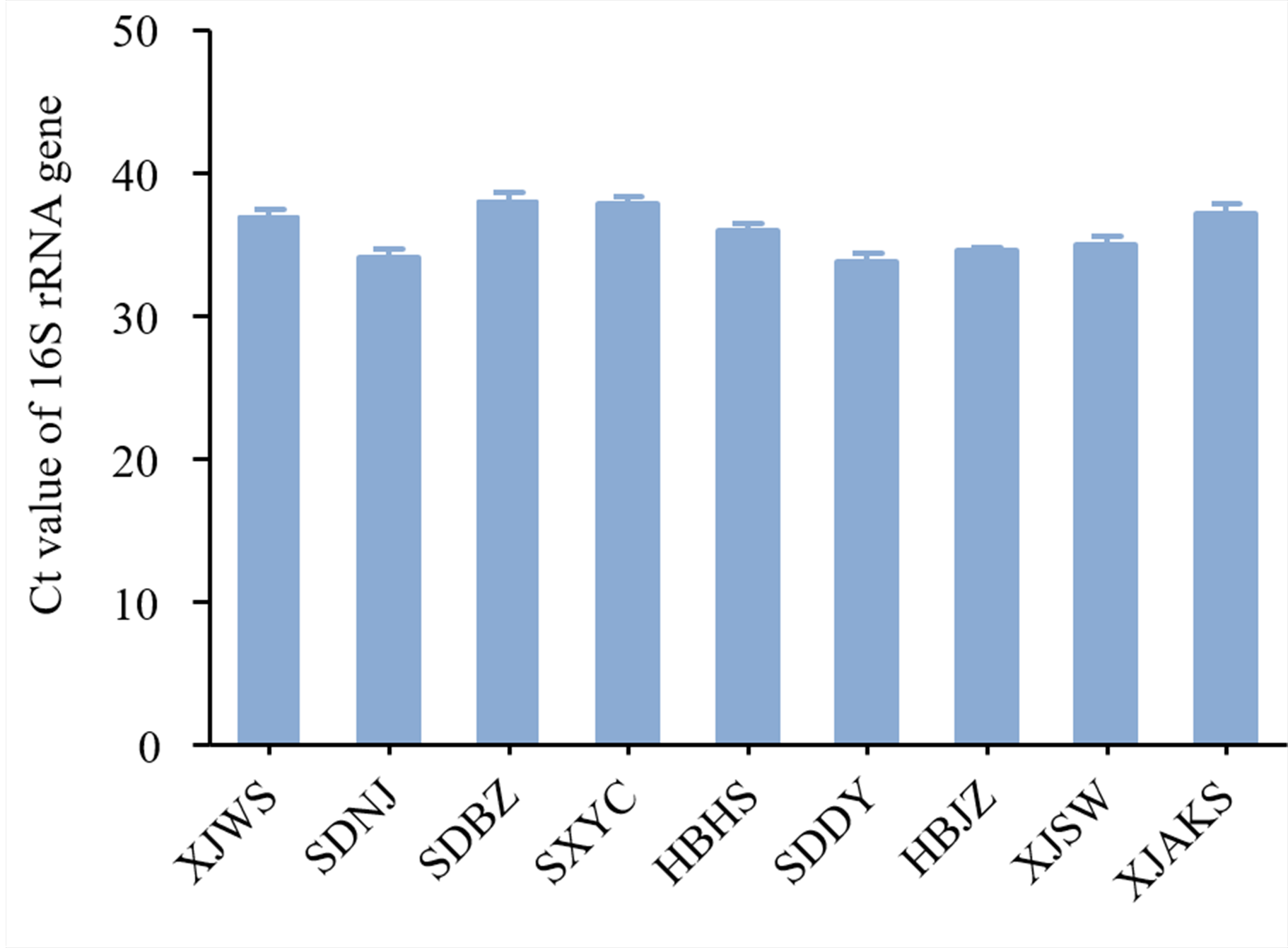

Supplement: Supplementary file 5 — Additional file 5: Fig. S4. The abundance of Sphingomonas in the gut of the IMI-S and IMI-R strains and SXYC, SDBZ, XJSW and HBHS field populations. The bars with lowercase letters (a, b, c) are significantly different according to one-way ANOVA, followed by Tukey's multiple comparison test (P< 0.05). [file 12915_2023_1586_MOESM5_ESM.tif]

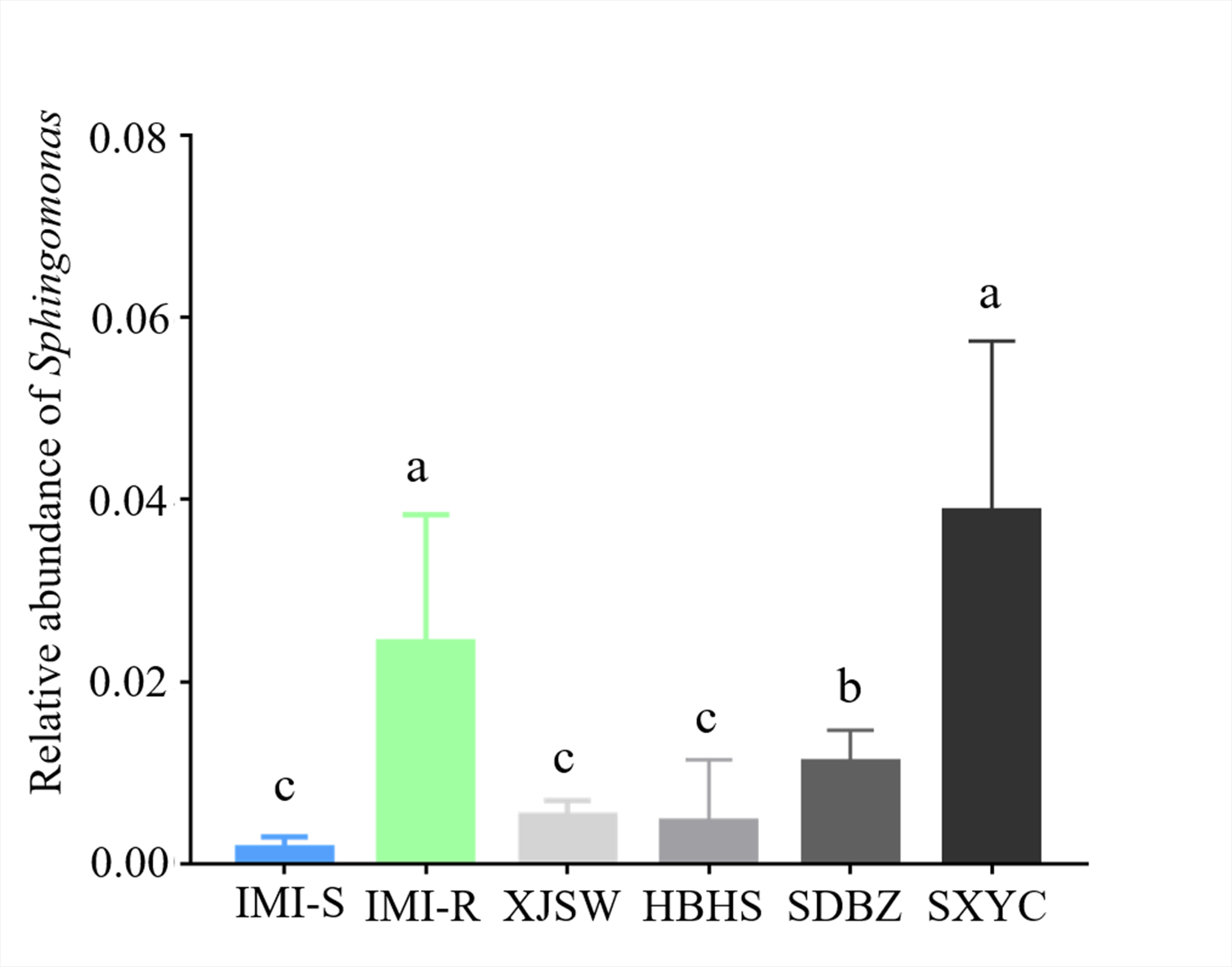

Supplement: Supplementary file 6 — Additional file 6: Fig. S5. The stand curve of IMI (A), urea IMI (B) and 5-OH IMI (C). [file 12915_2023_1586_MOESM6_ESM.tif]

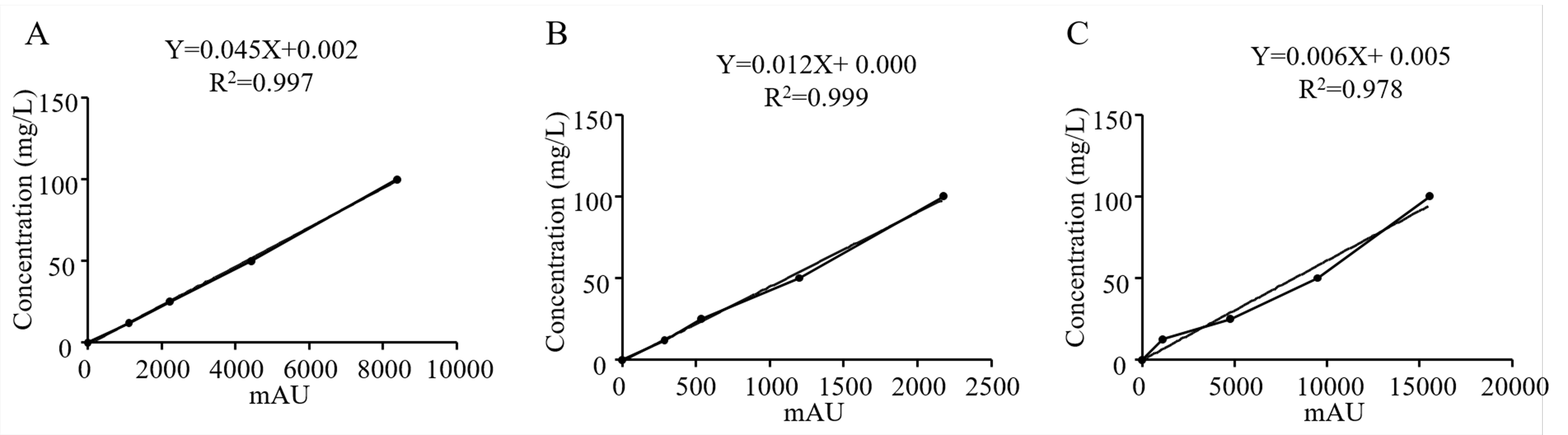

Supplement: Supplementary file 7 — Additional file 7: Fig. S6. The changes in OD600 value of Sphingomonas in the control group (A) and IMI group (B) during the 3-day cultivation. The bars with different lowercase letters (a, b, c) are significantly different (one-way ANOVA followed by Tukey's multiple comparison, P< 0.05). [file 12915_2023_1586_MOESM7_ESM.tif]
