# Supplementary material for: The gut symbiont Sphingomonas mediates imidacloprid resistance in the important agricultural insect pest Aphis gossypii Glover
Source: BMC Biol. 2023 Apr 17;21:86. doi: 10.1186/s12915-023-01586-2 (PMC10111731; doi:10.1186/s12915-023-01586-2)
Supplement: Supplementary file 8 — Additional file 8: Table S2. Collecting information of Aphis gossypii field populations. [file 12915_2023_1586_MOESM8_ESM.docx]

Table S2

Collecting information of *Aphis gossypii* field populations.

| NO. | Populations | Location (City, Province) | Longitude and latitude | Host plant | Year |
| --- | --- | --- | --- | --- | --- |
| 1 | SXYC | Yuncheng, Shanxi | 10.35° W, 34.45° N | cotton | 2020 |
| 2 | XJAKS | Aksu, Xinjiang | 40.55° W, 80.34° N | cotton | 2020 |
| 3 | SDDY | Dongying, Shandong | 118.49° W, 37.46° N | cotton | 2020 |
| 4 | HBHS | Hengshui, Hebei | 115.10° W, 37.33° N | cotton | 2020 |
| 5 | XJSW | Shawan, Xinjiang | 134.12° W, 48.15° N | cotton | 2020 |
| 6 | XJWS | Wusu, Xinjiang | 85.59° W, 43.41° N | cotton | 2020 |
| 7 | HBJZ | Jingzhou, Hubei | 111.20° W, 30.34° N | cotton | 2020 |
| 8 | SDJN | Jinan, Shandong | 116.55° W, 35.34° N | cotton | 2020 |
| 9 | SDBZ | Binzhou, Shandong | 36.55° W, 117.34° N | cotton | 2020 |
